# Airplane pilot mental health and suicidal thoughts: a cross-sectional descriptive study via anonymous web-based survey

**DOI:** 10.1186/s12940-016-0200-6

**Published:** 2016-12-15

**Authors:** Alexander C. Wu, Deborah Donnelly-McLay, Marc G. Weisskopf, Eileen McNeely, Theresa S. Betancourt, Joseph G. Allen

**Affiliations:** 1 Department of Environmental Health, Harvard T.H. Chan School of Public Health, 665 Huntington Avenue, Building 1, Room 1301, Boston, MA 02115 USA; 2 Department of Global Health and Population, Harvard T.H. Chan School of Public Health, 665 Huntington Avenue, Building 1, Room 1104, Boston, MA 02115 USA; 3 Harvard T.H. Chan School of Public Health, 401 Park Drive, Landmark Center, 404-L, Boston, MA 02215 USA

**Keywords:** Airline, Pilot, Mental health, Mental disorder, Depression, Suicidal

## Abstract

**Background:**

The Germanwings Flight 9525 crash has brought the sensitive subject of airline pilot mental health to the forefront in aviation. Globally, 350 million people suffer from depression–a common mental disorder. This study provides further information on this important topic regarding mental health especially among female airline pilots. This is the first study to describe airline pilot mental health–with a focus on depression and suicidal thoughts–outside of the information derived from aircraft accident investigations, regulated health examinations, or identifiable self-reports, which are records protected by civil aviation authorities and airline companies.

**Methods:**

This is a descriptive cross-sectional study via an anonymous web-based survey administered between April and December 2015. Pilots were recruited from unions, airline companies, and airports via convenience sampling. Data analysis included calculating absolute number and prevalence of health characteristics and depression scores.

**Results:**

One thousand eight hundred thirty seven (52.7%) of the 3485 surveyed pilots completed the survey, with 1866 (53.5%) completing at least half of the survey. 233 (12.6%) of 1848 airline pilots responding to the Patient Health Questionnaire 9 (PHQ-9), and 193 (13.5%) of 1430 pilots who reported working as an airline pilot in the last seven days at time of survey, met depression threshold–PHQ-9 total score ≥ 10. Seventy-five participants (4.1%) reported having suicidal thoughts within the past two weeks. We found a significant trend in proportions of depression at higher levels of use of sleep-aid medication (trend test z = 6.74, *p* < 0.001) and among those experiencing sexual harassment (z = 3.18, *p* = 0.001) or verbal harassment (z = 6.13, *p* < 0.001).

**Conclusion:**

Hundreds of pilots currently flying are managing depressive symptoms perhaps without the possibility of treatment due to the fear of negative career impacts. This study found 233 (12.6%) airline pilots meeting depression threshold and 75 (4.1%) pilots reporting having suicidal thoughts. Although results have limited generalizability, there are a significant number of active pilots suffering from depressive symptoms. We recommend airline organizations increase support for preventative mental health treatment. Future research will evaluate additional risk factors of depression such as sleep and circadian rhythm disturbances.

**Electronic supplementary material:**

The online version of this article (doi:10.1186/s12940-016-0200-6) contains supplementary material, which is available to authorized users.

## Background

On March 24, 2015, Germanwings flight 4U 9525 crashed into the French Alps killing 150 people. Investigators of this tragic event report the 27-year-old co-pilot deliberately crashed the plane [1, 2]. Further examination of the co-pilot’s history found evidence suggesting the co-pilot suffered from clinical depression [3]. Previous suicide attempts and having a history of mental disorders, particularly clinical depression, are risk factors of suicide [4].

Clinical depression, also referred to as major depressive disorder (MDD) – characterized by at least two weeks of depressed mood or loss of interest along with at least four additional symptoms of depression [5] – is the second leading cause of years of life lived with a disability (YLDs) [6] and third most important cause of disease burden worldwide and affects an estimated 350 million people [7, 8]. The United States (U.S.) leads the world in the percent of people (21%) who will have a mood disorder, including MDD, over their lifetime [9]. In the U.S., more females report having depression than males [8], 17% of people will have MDD over their lifetime [10], and 7% will have experienced an MDD in the past year [11]. MDD symptoms cause significant distress and social, occupational, and life activity impairment and may first appear at any age with most new cases occurring around 20 years of age in the U.S [5, 12]. Estimates of prevalence of diagnosed MDD or depressive symptoms related to MDD among high stress occupations include 12% among deployed and 13% among previously deployed U.S. military personnel [13], 7% among U.S. emergency medical technicians [14], and 10–17% among U.S. police officers [15].

There are an estimated 140,000 airline pilots internationally with about 70,000 in the U.S [16]. The majority of pilots are male: just over 4% of all pilots are female [17]. In the U.S., the Department of Transportation, Federal Aviation Administration (FAA) sets requirements for aeromedical examiners (AMEs) to evaluate fitness of pilots. Only through self-disclosure are mental disorders discussed and noted in pilot health records; AMEs do not diagnose mental health conditions (See Additional file 1 for more details). Underreporting of mental health symptoms and diagnoses is probable among airline pilots due to the public stigma of mental illness and fear among pilots of being “grounded” or not fit for duty [18, 19].

Studies of airline pilots have either not anonymously assessed mental health or had limitations in doing so. Prior studies have found a lower prevalence of depression among military pilots [19] and airline pilots [18, 20] compared to the general population. However, there is concern about underreporting of adverse health symptoms and incomplete medical information due to pilots protecting professional interests [18], and underreporting of the use of antidepressants in aeromedical examinations has been found among a group of U.S. civilian pilots involved in fatal accidents [21]. Anonymous surveying could alleviate some of the issues with underreporting. Only one study of airline pilots has used anonymous reporting, but it did not specifically evaluate depression nor suicidal thoughts [18].

The objective of our study was to provide a more accurate description of mental health among commercial airline pilots underscoring symptoms related to clinical depression (hereafter also referred to as “depression”) using an anonymous survey to guard against fears of stigma and job discrimination. This study did not conduct clinical interviews of survey respondents to confirm diagnosis of depression, nor did it have access to medical records. This is the first study that we are aware of to describe mental health in a convenience sample of pilots outside of the information derived from aircraft accident investigations [22] or regulated health examinations, which are identifiable self-reports and physician interviews, and are records protected by civil aviation authorities and airline companies.

## Methods

### Study design

This is a cross-sectional descriptive study of commercial airline pilots who completed a web-based survey between April and December 2015. In order to protect pilots’ ability to hold an FAA Medical Certificate the survey was completely anonymous and no internet protocol (IP) addresses were collected. The Institutional Review Board of the Harvard T.H. Chan School of Public Health reviewed and exempted the study.

Recruitment methods included targeted e-mail, newsletters, word-of-mouth, handing postcards to pilots, and aviation publication advertisements. Airline pilot populations that gave rise to the survey population included pilot unions (>5 unions), airline representatives (>65 airlines), pilot groups (>12 groups), and aviation safety organizations (>2 organizations). We targeted female pilots in recruiting because of the small percentage of female pilots among the general airline pilot population. We downloaded 3485 surveys on December 31, 2015.

The data analysis included all answered questions. We defined a completed survey as answering the final non-optional question. We assumed each participant was a pilot and only completed one survey. Several questions in the survey require knowledge that would only be readily available to pilots. An active pilot (co-author DDM) reviewed surveys for potential non-pilot participants. All surveys passed this screening. Finally, the survey instructed participants to leave the checkbox unmarked if they did not have a diagnosis of the listed disorder.

### Survey description

The survey utilized standardized questions from the Job Content Questionnaire [23] and the Centers for Disease Control – National Center for Health Statistics (CDC-NCHS) National Health and Nutrition Examination Survey (NHANES) (CDC-NCHS 2011–2012) [24], which previous researchers applied to evaluate U.S. flight attendant health [25]. Participants were not likely to be biased to mental health outcomes since the survey covered other work and health topics. The survey took about 30 min for completion. We utilized Qualtrics software (Qualtrics, Provo, Utah) to disseminate surveys and collect responses.

### Statistical analysis

We utilized STATA software (Version 13.1, StataCorp, College Station, Texas) for data analysis. We applied two-sided unequal variances t-test for continuous variables, Pearson’s chi-squared test or Fisher’s exact test (*n* ≤ 5) for categorical variables, and nonparametric test for trend across ordered groups. Age was categorized into four groups using quartiles. We utilized the Kruskal-Wallis equality-of-populations rank test to compare scores among age categories. Significance was defined as *p*-value <0.05.

### Outcome assessment

We evaluated depressive symptoms via the Patient Health Questionnaire (PHQ-9) depression module utilized in previous NHANES surveys (e.g., NHANES 2005–2006 and 2011–2012), which is well validated and used in clinical studies assessing depression [26, 27]. Briefly, the PHQ-9 depression module asks nine questions, which are the nine criteria for diagnosing depressive disorder in the *Diagnostic and Statistical Manual of Mental Disorders, Edition 4* (DSM-IV) [26]. Researchers record scores as frequency of depression symptoms over the past two weeks [26]. Response categories include “not at all,” “several days,” “more than half the days” and “nearly every day” and given a score ranging from 0 to 3, respectively. Total summed scores per participant range from 0 to 27. Studies evaluating validity of PHQ-9 report a total score of 10 or greater had an 88% sensitivity and 88% specificity for depression [26] with a kappa of 0.56 to 0.74 between PHQ-9 diagnosis and diagnosis by an independent mental health professional [28, 29]. Therefore, we refer to meeting the cut-off of having a PHQ-9 total score of 10 as depression.

## Results

Of the 3485 participants, 1837 (52.7%) completed the survey and 1866 (53.5%) answered at least half of the survey. Completers initiated surveys from over 50 countries. Major locations included the United States (1586, 45.5%), Canada (438, 12.6%), and Australia (387, 11.1%). Participants initiated surveys in Europe (406, 11.7%), Asia (413, 11.9%), South America (165, 4.7%), and South Africa (8, 0.2%). Locations with the most participants in Europe were Spain (134, 3.9%), United Kingdom (65, 1.9%), and Germany (32, 0.9%). For Asia the locations were United Arab Emirates (172, 4.9%), Hong Kong (147, 4.2%), and Thailand (13, 0.4%). For South America the locations were Colombia (74, 2.1%), Brazil (71, 2.0%), and Chile (8, 0.23%). Due to many missing responses among non-completers, comparisons between completers and non-completers were constrained to average tenure as a pilot (completers 18.0 years, 95% CI 17.5 to 18.4 vs. non-completers 16.9, 16.2 to 17.6, *p* = 0.012) and the proportion working one trip as an airline pilot in the past 30 days (completers 1417, 77.1% vs. non-completers 1099, 73.0%, *p* = 0.006). The response rate [30] among those who answered at least one question was 0.68. Of the 1826 who provided age, about half were middle aged with the median age for females and males at 42 (IQR 36–51) and 50 (IQR 41–60) years, respectively. Half of participants worked at least 16 years as a pilot and nearly four out of five worked one trip as an airline pilot in the past 30 days. The majority of respondents were non-smokers, married, and white. Over 60% earned a four-year college degree or had graduate education (Table 1).

**Table 1:** Characteristics of survey participants by age

	Age <41 n (%)	Age 41 to 50 n (%)	Age 51 to 60 n (%)	Age >60 n (%)	Total n (%)
Participants who indicated age	489 (26.8)	493 (27.0)	449 (24.6)	395 (21.6)	1826 (100%)
Gender (*n* = 1826)^a^					
Female	114 (23.3)	71 (14.4)	56 (12.5)	9 (2.3)	250 (13.7)
Male	375 (76.7)	422 (85.6)	393 (87.5)	386 (97.7)	1576 (86.3)
Tenure (*n* = 1826)^a^					
< 6 years	165 (33.7)	17 (3.5)	8 (1.8)	1 (0.3)	191 (10.5)
6–10 years	206 (42.1)	94 (19.1)	15 (3.3)	4 (1.0)	319 (17.5)
11–15 years	100 (20.5)	140 (28.4)	56 (12.5)	12 (3.0)	308 (16.9)
16–20 years	18 (3.7)	168 (34.1)	85 (18.9)	30 (7.6)	301 (16.5)
21–25 years	0 (0.0)	68 (13.8)	110 (24.5)	43 (10.9)	221 (12.1)
> 25 years	0 (0.0)	6 (1.2)	175 (39.0)	305 (77.2)	486 (26.6)
Recently Worked^b^ (*n* = 1826)^a^					
No	20 (4.1)	31 (6.3)	53 (11.8)	308 (78.0)	412 (22.6)
Yes	469 (95.9)	462 (93.7)	396 (88.2)	87 (22.0)	1414 (77.4)
Education (*n* = 1817)^a^					
Less than high school diploma	2 (0.4)	1 (0.2)	3 (0.7)	2 (0.5)	8 (0.4)
High school or GED	60 (12.4)	72 (14.7)	49 (10.9)	38 (9.6)	219 (12.1)
Some college, no degree	85 (17.6)	82 (16.7)	53 (11.8)	81 (20.5)	301 (16.6)
Two-year college degree	62 (12.8)	47 (9.6)	27 (6.0)	26 (6.6)	162 (8.9)
Four-year college degree	217 (44.9)	221 (45.0)	226 (50.5)	148 (37.5)	812 (44.7)
Graduate education	57 (11.8)	68 (13.9)	90 (20.1)	100 (25.3)	315 (17.3)
Marital Status (*n* = 1817)^a^					
Married	274 (56.7)	395 (80.5)	356 (79.5)	325 (82.3)	1350 (74.3)
Widowed	1 (0.2)	0 (0.0)	3 (0.7)	14 (3.5)	18 (1.0)
Divorced	11 (2.3)	35 (7.1)	42 (9.4)	29 (7.3)	117 (6.4)
Separated	4 (0.8)	12 (2.4)	4 (0.9)	8 (2.0)	28 (1.5)
Never Married	106 (22.0)	23 (4.7)	20 (4.5)	8 (2.0)	157 (8.6)
Living with partner	87 (18.0)	26 (5.3)	23 (5.1)	11 (2.8)	147 (8.1)

^a^Two-sided Fisher’s exact test *p*-value <0.01

^b^Recently worked means worked one trip as an airline pilot in past 30 days

**Fig. 1:**
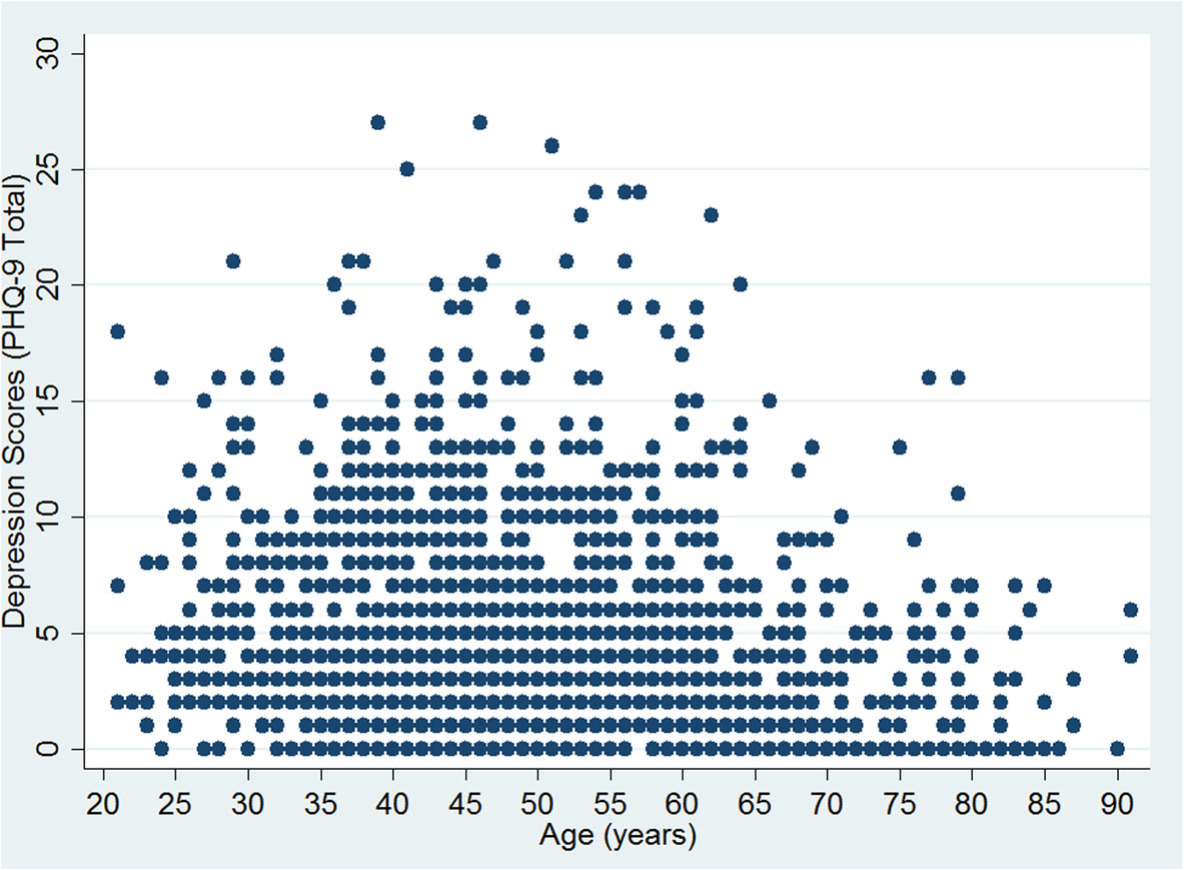
Total Depression Scores by Age (*n* = 1848). Each dot represents one participant. Some dots overlap

**Fig. 2:**
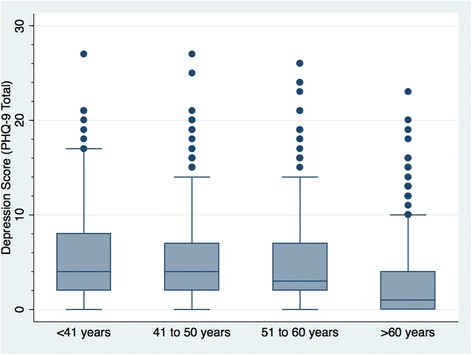
Total Depression Scores by Age Quartiles (years) (*n* = 1848). Each dot represents an outlier. Maximum possible depression score (PHQ-9 Total) is 27

Nearly all ages up to 80 years had pilots who met depression threshold–PHQ-9 total score ≥ 10 (Fig. 1). Among age categories, median total depression score decreased with increasing age quartile (Kruskal-Wallis rank test chi-square with ties = 157.63 with 3 d.f., *p* < 0.001) (Fig. 2). The number of pilots self-reporting having at least one day of poor mental health during the past month ranged from 94 (26.9%) among those over age 60 to 273 (56.5%) among 41 to 50 years (Table 2). Forty-seven (9.6%) respondents up to age 40 and 110 (11.9%) age 41 to 60 years reported having at least eight days of poor mental health during the past month (Table 2). Females had a greater proportion of having at least one day of poor mental health during the past month (females 139, 55.2% vs. males 697, 45.6%, *p* = 0.005) or having ever been diagnosed with depression (females 12, 4.7% vs. males 46, 2.9%, *p* = 0.12).

**Table 2:** Mental health characteristics of survey participants

	Age <41 n (%)	Age 41 to 50 n (%)	Age 51 to 60 n (%)	Age >60 n (%)	Total n (%)
Mental Health not Good (Days/Past month) (*n* = 1759)^a^					
0 days	230 (47.4)	210 (43.5)	236 (53.5)	256 (73.1)	932 (53.0)
1–7 days	208 (42.9)	215 (44.5)	153 (34.7)	66 (18.9)	642 (36.5)
8–14 days	19 (3.9)	24 (5.0)	20 (4.5)	11 (3.1)	74 (4.2)
15–21 days	20 (4.1)	23 (4.8)	10 (2.3)	4 (1.1)	57 (3.2)
22–28 days	3 (0.6)	3 (0.6)	4 (0.9)	4 (1.1)	14 (0.8)
> 28 days	5 (1.0)	8 (1.7)	18 (4.1)	9 (2.6)	40 (2.3)
Ever diagnosed with sleep disorder (*n* = 1826)^ab^	15 (3.1)	40 (8.1)	54 (12.0)	51 (12.9)	160 (8.8)
Ever diagnosed with depression (*n* = 1826)^b^	11 (2.3)	15 (3.0)	11 (2.5)	20 (5.1)	57 (3.1)
In the past 7 days, how many days did you experience the following symptoms?					
Fatigue, Everyday (*n* = 1826)	15 (3.1)	26 (5.3)	30 (6.7)	17 (4.3)	88 (4.8)
Depression, Everyday (*n* = 1826)	4 (0.8)	5 (1.0)	7 (1.6)	3 (0.8)	19 (1.0)

^a^Two-sided Chi-square or Fisher’s Exact Test *p*-value < 0.05

^b^Including unknown number of participants that did not answer

Median PHQ-9 total scores was lowest among those over 60 (Table 3) and were the same among sex with males (3, IQR(1–7), *n* = 1591) having a greater spread of total scores than females (3, (2–6), *n* = 255). The greatest differences in proportions between females and males experiencing at least one day of problems were among PHQ-9 items #1: Having little interest or pleasure in doing things (females 87, 34.1% vs. males 683, 43.1%, *p* = 0.007) and #5: Having poor appetite or overeating (140, 55.1% vs. 687, 43.5%, *p* = 0.001).

**Table 3:** Patient Health Questionnaire Depression Module (PHQ-9) by Age Category

Over the last 2 weeks, how often have you been bothered by the following problems:	Age <41 n (%)	Age 41 to 50 n (%)	Age 51 to 60 n (%)	Age >60 n (%)	Total n (%)
1) Little interest or pleasure in doing things? (*n* = 1812)^a^ ^b^					
Not at all	247 (50.6)	258 (52.6)	265 (59.4)	282 (72.9)	1052 (58.1)
Several days	189 (38.7)	184 (37.5)	138 (30.9)	80 (20.7)	591 (32.6)
More than half the days	39 (8.0)	33 (6.7)	21 (4.7)	17 (4.4)	110 (6.1)
Nearly everyday	13 (2.7)	16 (3.3)	22 (4.9)	8 (2.1)	59 (3.3)
2) Feeling down, depressed, or hopeless? (*n* = 1800)^a^ ^b^					
Not at all	344 (70.8)	349 (71.1)	341 (76.8)	323 (85.2)	1357 (75.4)
Several days	113 (23.3)	111 (22.6)	75 (16.9)	44 (11.6)	343 (19.1)
More than half the days	22 (4.5)	24 (4.9)	17 (3.8)	7 (1.9)	70 (3.9)
Nearly everyday	7 (1.4)	7 (1.4)	11 (2.5)	5 (1.3)	30 (1.7)
3) Trouble falling or staying asleep, or sleeping too much? (*n* = 1809)^a^ ^b^					
Not at all	100 (20.6)	109 (22.3)	122 (27.3)	219 (56.6)	550 (30.4)
Several days	241 (49.6)	245 (50.1)	192 (43.0)	106 (27.4)	784 (43.3)
More than half the days	95 (19.6)	85 (17.4)	83 (18.6)	34 (8.8)	297 (16.4)
Nearly everyday	50 (10.3)	50 (10.2)	50 (11.2)	28 (7.2)	178 (9.8)
4) Feeling tired or having little energy? (*n* = 1806)^a^ ^b^					
Not at all	79 (16.2)	77 (15.8)	107 (23.9)	195 (51.2)	458 (25.4)
Several days	267 (54.7)	261 (53.4)	231 (51.6)	141 (37.0)	900 (49.8)
More than half the days	101 (20.7)	104 (21.3)	74 (16.5)	28 (7.4)	307 (17.0)
Nearly everyday	41 (8.4)	47 (9.6)	36 (8.0)	17 (4.5)	141 (7.8)
5) Poor appetite or overeating? (*n* = 1805)^a^ ^b^					
Not at all	223 (46.0)	229 (46.9)	247 (55.3)	290 (75.3)	989 (54.8)
Several days	158 (32.6)	163 (33.4)	137 (30.7)	69 (17.9)	527 (29.2)
More than half the days	73 (15.1)	72 (14.8)	42 (9.4)	16 (4.2)	203 (11.3)
Nearly everyday	31 (6.4)	24 (4.9)	21 (4.7)	10 (2.6)	86 (4.8)
6) Feeling bad about yourself – or that you are a failure or have let yourself or your family down? (*n* = 1811)^a^ ^b^					
Not at all	345 (70.7)	353 (72.0)	345 (77.4)	326 (84.2)	1369 (75.6)
Several days	106 (21.7)	103 (21.0)	72 (16.1)	50 (12.9)	331 (18.3)
More than half the days	30 (6.2)	23 (4.7)	16 (3.6)	6 (1.6)	75 (4.1)
Nearly everyday	7 (1.4)	11 (2.2)	13 (2.9)	5 (1.3)	36 (2.0)
7) Trouble concentrating on things, such as reading the newspaper or watching TV? (*n* = 1803)^a^ ^b^					
Not at all	305 (62.5)	287 (58.5)	304 (68.3)	305 (80.5)	1201 (66.6)
Several days	149 (30.5)	167 (34.0)	107 (24.0)	62 (16.4)	485 (26.9)
More than half the days	24 (4.9)	29 (5.9)	18 (4.0)	7 (1.9)	78 (4.3)
Nearly everyday	10 (2.1)	8 (1.6)	16 (3.6)	5 (1.3)	39 (2.2)
8) Moving or speaking so slowly that other people could have noticed? Or the opposite – being so fidgety or restless that you have been moving around a lot more than usual? (*n* = 1810)^a^ ^b^					
Not at all	414 (84.8)	407 (83.1)	390 (87.8)	357 (92.0)	1568 (86.6)
Several days	56 (11.5)	66 (13.5)	33 (7.4)	23 (5.9)	178 (9.8)
More than half the days	14 (2.9)	13 (2.7)	12 (2.7)	7 (1.8)	46 (2.5)
Nearly everyday	4 (0.8)	4 (0.8)	9 (2.0)	1 (0.3)	18 (1.0)
9) Thoughts that you would be better off dead or of hurting yourself in some way? (*n* = 1798)					
Not at all	469 (96.1)	468 (95.7)	426 (96.0)	360 (95.5)	1723 (95.8)
Several days	13 (2.7)	16 (3.3)	13 (2.9)	14 (3.7)	56 (3.1)
More than half the days	4 (0.8)	3 (0.6)	1 (0.2)	1 (0.3)	9 (0.5)
Nearly everyday	2 (0.4)	2 (0.4)	4 (0.9)	2 (0.5)	10 (0.6)
Median PHQ-9 total score (IQR) n	4 (2–8) 489	4 (2–7) 491	3 (2–7) 449	1 (0–4) 389	3 (2–7) 1848
How difficult have these problems made it for you to do your work, take care of things at home, or get along with people? (*n* = 1812)^a^ ^b^					
Not at all	269 (55.1)	262 (53.5)	282 (62.8)	306 (79.5)	1119 (61.8)
Somewhat	199 (40.8)	207 (42.2)	139 (31.0)	70 (18.2)	615 (33.9)
Very	17 (3.5)	16 (3.3)	17 (3.8)	5 (1.3)	55 (3.0)
Extremely	3 (0.6)	5 (1.0)	11 (2.5)	4 (1.0)	23 (1.3)

^a^Two-sided Chi-square or Fisher’s Exact Test *p*-value < 0.05

^b^Non-parametric test for trend of item scores across age groups, *p*-value < 0.05

Among participants who answered the PHQ-9 questions, 233 (12.6%) met threshold associated with clinical levels of depression. Two-hundred-and-four (12.8%) males and 29 (11.4%) females (χ^2^ *p* = 0.52) met depression threshold. One-hundred and ninety-two (13.6%) of the 1413 pilots who reported working as an airline pilot in the last 30 days met depression threshold (Table 4).

**Table 4:** Depression among survey participants by age group

Worked as an airline pilot in past 7 days (*n* = 1199)					
Elevated Depression Symptoms^a^	Age <41	Age 41 to 50	Age 51 to 60	Age >60	Total
No, n (%)	350 (85.2)	339 (85.4)	281 (87.5)	63 (90.0)	1033 (86.2)
Yes, n (%)	61 (14.8)	58 (14.6)	40 (12.5)	7 (10.0)	166 (13.8)
Worked one trip as an airline pilot in past 30 days (*n* = 1413)					
Elevated Depression Symptoms^a^	Age <41	Age 41 to 50	Age 51 to 60	Age >60	Total
No, n (%)	401 (85.5)	392 (85.0)	350 (88.4)	78 (89.7)	1221 (86.4)
Yes, n (%)	68 (14.5)	69 (15.0)	46 (11.6)	9 (10.3)	192 (13.6)

^a^Elevated Depression Symptoms means PHQ-9 total score ≥ 10

Among screening questions concerning psychological symptoms (PHQ-9 questions 1,2,6,7,9), a greater proportion of males than females reported “nearly every day” experiences in loss of interest (males 59, 3.7% vs. females 2, 0.8%, *p* = 0.01), feeling depressed (27, 1.7% vs. 4, 1.6%, *p* = 1.00), feeling like a failure (34, 2.2% vs. 3, 1.2%, *p* = 0.47), trouble concentrating (34, 2.2% vs. 5, 2.0%, *p* = 1.00), and thinking they would be better off dead or having thoughts of self-harm (10, 0.6% vs. 0, 0.0%, *p* = 0.37). Seventy-five participants (4.1%) reported having thoughts of being better off dead or self-harm within the past two weeks (pilots working within the past month 49, 3.5% vs. not 26, 6.4%, *p* = 0.008). A higher percentage of males (23, 1.5%) vs. females (1, 0.4%, *p*= 0.24) felt that the problems they reported on the PHQ-9 made it very or extremely difficult for them to work, take care of home matters, or engage in healthy relationships with people.

Among those who answered the PHQ-9 questions and indicated their location of initiating the survey, 232 (12.6%) met threshold associated with clinical levels of depression. We stratified location of survey initiation into countries exhibiting more western cultural influence (i.e., countries in North and South America, Europe, or Australia) and those exhibiting less (i.e., countries in Asia). There were 1576 (85.5%) participants initiating surveys in more culturally western countries and 267 (14.5%) in less culturally western countries. Countries with more western cultural influence had a lower percentage of pilots meeting depression threshold than others (172, 10.9% vs. 60, 22.5%, *p* < 0.001). Examining this further by sex reveals the prevalence of meeting threshold is similar among females (more western 27, 11.3% vs. less 2, 13.3%, *p* = 0.68). This was not the case among males with more western having a prevalence lower than less western (145, 10.9% vs. 58, 23.0%, *p* < 0.001). Furthermore, 61 participants (3.9%) initiating surveys in more culturally western countries compared to 14 (5.3%) in less western countries reported having thoughts of being better off dead or self-harm within the past two weeks. This difference was not statistically significant (*p* = 0.31). Grouped by sex, there was no significant difference among females (more western 7, 2.9% vs. less 0, 0.0%, *p* = 1.00) or males (more western 54, 4.1% vs. less 14, 5.6%, *p* = 0.31).

The proportion meeting depression threshold among pilots working in the past month was higher as the frequency of taking sleep aid medicines in the past month increased (Table 5). The survey found 19 (16.2%) working pilots met depression threshold among those consuming more than one drink of alcohol per day. The proportion of pilots meeting the same threshold was higher among those experiencing sexual harassment (36.4% among those experiencing harassment 4 or more times in the past week) or verbal harassment (42.9% among those experiencing harassment 4 or more times in the past week) in the last 12 months at work.

**Table 5:** Sleep aid medicine use, alcohol consumption, sexual harassment, verbal harassment and depression among working airline pilots

Taken medicine (prescribed or “over the counter”) to help with sleep among those worked in past 30 days (*n* = 1425)^a^					
Elevated Depression Symptoms^b^	Not during past month	< Once a week	Once or twice a week	Three or more times a week	Total
No, n (%)	875 (89.6)	170 (86.7)	118 (79.7)	70 (66.7)	1233 (86.5)
Yes, n (%)	101 (10.4)	26 (13.3)	30 (20.3)	35 (33.3)	192 (13.5)
Alcohol Consumption among those worked in past 30 days (*n* = 1417)					
Elevated Depression Symptoms^b^	Never or < 1 Drink per month	>1 Drink per month to 1 Drink per week	> 1 Drink per week to 1 Drink per day	>1 Drink per day	Total
No, n (%)	119 (83.8)	341 (87.0)	666 (87.0)	98 (83.8)	1224 (86.4)
Yes, n (%)	23 (16.2)	51 (13.0)	100 (13.1)	19 (16.2)	193 (13.6)
Frequency of sexual harassment in last 12 months among those worked in past 30 days (*n* = 1414)^a^					
Elevated Depression Symptoms^b^	Never	1 time	2–3 times	4 or more times	Total
No, n (%)	1129 (87.0)	62 (88.6)	24 (68.6)	7 (63.6)	1222 (86.4)
Yes, n (%)	169 (13.0)	8 (11.4)	11 (31.4)	4 (36.4)	192 (13.6)
Frequency of verbal harassment in last 12 months among those worked in past 30 days (*n* = 1398)^a^					
Elevated Depression Symptoms^b^	Never	1 time	2–3 times	4 or more times	Total
No, n (%)	973 (88.7)	148 (83.6)	74 (71.8)	12 (57.1)	1207 (86.3)
Yes, n (%)	124 (11.3)	29 (16.4)	29 (28.2)	9 (42.9)	191 (13.7)

^a^Fisher’s exact test (two-sided) and trend test *p*-value < 0.05

^b^Elevated Depression Symptoms means PHQ-9 total score ≥ 10

## Discussion

The Germanwings crash in March of 2015 has brought a sensitive subject to the forefront in aviation; pilot mental health. To date, this is the first study providing a description from anonymous reporting of mental health among commercial airline pilots with an emphasis on depression and suicidal thoughts. Our study also oversampled female pilots (13.7% of our study population) to better describe this minority population (about 4%) among commercial airline pilots [17]. We utilized an anonymous web-based survey to collect responses and a clinically validated questionnaire, PHQ-9, to determine depression (PHQ-9 total score ≥ 10).

In the context of reporting depression, female pilots reported more days with poor mental health and having more diagnosed depression than male pilots, which mirrors reporting among the general population. The prevalence of depression (12.6%) among pilots from our study is much higher than some studies utilizing identifiable surveys and medical records [19, 20] and possibly lower than another study [31]. One study utilizing anonymous case reporting among commercial airline pilots between years 1996 and 1999 found the prevalence of psychiatric disease around 7.5% [18]. However, this study did not report information on depression or suicidal thoughts and its authors acknowledged the inability to identify an exact reference population [18]. In addition, a study utilizing the medical record database of U.S. Air Force pilots estimated a prevalence of depression of 0.06% during years 2001–2006 [19]. Researchers evaluating airline pilots in the New Zealand Health Survey found a prevalence of depression of 1.9% during years 2009–2010 [20]. A report on Air Canada pilots with long term disability found a prevalence of mental disorders at 15.8% [31]. These studies did not evaluate prevalence of pilots having suicidal thoughts. Furthermore, estimates of prevalence of depression or depressive symptoms among other high stress occupations include 12% among deployed and 13% among previously deployed U.S. military personnel [13], 7% among U.S. emergency medical technicians [14], and 10–17% among U.S. police officers [15]. From these studies of mental illness in pilots and similar high stress occupations, the prevalence of depression in our results seem probable. Moreover, the higher prevalence of depression among victims of frequent sexual or verbal harassment in our study provides further evidence of its existence among airline pilots, deep negative effects on its victims, and the urgent need to eliminate this form of harassment and help this subpopulation of workers.

Our study found 75 pilots (4.1%) reported having thoughts of being better off dead or self-harm within the past two weeks. To our knowledge, this is the most current measure of the prevalence of suicidal thoughts among airline pilots. One study estimated an aircraft assisted suicide rate of 0.33% over a 20 year period in the U.S. following analysis of aircraft accidents from 1956 to 2012 [32]. However, this study measured completed suicides, not prevalence of suicidal thoughts.

We hypothesize two possible explanations for the lower prevalence of meeting depression threshold in pilots who initiated the survey in more western culture countries compared to others. One reason is the type of culture the pilots identify themselves with and country of survey initiation is not an accurate match. If true more western culture pilots were flying longer trips (such as from western to eastern culture countries) compared to true less western culture pilots, then these more western culture pilots may be more likely to initiate surveys in less western culture countries because of more downtime between flights. This could result in the misclassification of less western culture pilots appearing to have higher prevalence of meeting threshold for depression. Underlying factors could stem from longer trips increasing the risk of experiencing greater circadian rhythm disruption and longer exposure to other possible occupational factors related to mental illness. This misclassification also could occur the other way with healthier true less western culture pilots flying to more western culture countries and initiating surveys. Thus making western pilots appear healthier.

Another explanation for this result is that type of culture the pilots identify themselves with and country of survey initiation is an accurate match and that pilots from more western culture countries in our study have a lower prevalence of meeting depression threshold. We were unable to validate what culture pilots identify with due to lack of data. Nevertheless, even if the country of survey initiation accurately matches with pilots’ culture identification, our study has limited data on pilots surveyed outside western culture countries.

The prevalence of having suicidal thoughts between more western and less western culture countries of survey initiation was not significantly different at the 0.05 level. That said, the slightly higher prevalence of suicidal thoughts among less western culture countries may be due to the reasons given for the difference in prevalence of depression.

Additionally, the results of the comparison of more against less western culture countries in our study do not align with patterns in survey results of mental disorders around the world [33]. These surveys find more western culture countries generally having a higher 12-month prevalence of mood disorders [33]. However, researchers note that differences in mood disorder prevalence between high and low prevalence countries are likely smaller than the surveys show [33]. This is likely due to more underestimation of prevalence in low prevalence countries [33]. Consequently, this provides further evidence that the type of culture the pilots identify with in our study and country of survey initiation is not an accurate match.

Moving more generally, the topic of mental illness among airline pilots is not new, but identifying and assisting pilots with mental illness remains a present day challenge. Although the results of this study do not gauge pilots’ level of access to mental health treatment, it stimulates dialogue of treatment options available to assist pilots. More importantly, the subpopulations of victims of sexual or verbal harassment need even more urgent assistance. That said, barriers to seeking treatment for mental health issues among high stress occupations such as military personnel deployed in combat operations, emergency situation first responders, and firefighters and police officers are documented in the literature [34–36]. Although different in degree and severity of stressors, commercial airline pilots may experience similar occupational and individual barriers to seeking treatment [37]. These include shift-work, long and continuous hours, and increased stigma towards admitting one has mental health problems resulting from work.

Long and continuous work-hours make scheduling treatment difficult [38]. In addition, researchers attribute stigma among workers in high stress public safety protection occupations, which we argue includes piloting commercial aircraft, to the emphasis on being resilient and independent; thus, admitting having a mental health problem is extremely difficult [39, 40]. Other barriers to seeking treatment include increased social withdrawal among those experiencing symptoms of mental health problems such as depression [41] and concerns toward treatment (e.g., not trusting mental health professionals) [41, 42] and self-reporting (e.g., belief admitting will cause harm to career) [43], and social norms (e.g., weak support of those getting treatment) [34].

Since mental health problems are prevalent among our participants and maybe exacerbated in high stress work situations, we agree with the argument that organizations are responsible for ensuring employees who develop mental health problems receive timely mental health treatment [40]. Houdmont, Leka, and Sinclair [34] discuss three ways to increase treatment seeking among employees: (1) normalizing the receipt of needed mental health treatment (e.g., getting leadership endorsement), (2) emphasizing getting mental health treatment will prevent more severe problems from affecting employee performance, and (3) tailoring treatment to the occupational context. There are a number of deliverable solutions currently in place, which incorporate elements of these three recommendations.

Specifically, applying traditional cognitive behavioral treatment (CBT) while integrating work experiences shows promise in faster return to work among those on leave for mental health issues [44]. Furthermore, research supports the efficacy of internet-based treatments (e.g., CBT delivered online) as a viable option [45] for mild to moderate depression [46]. Reviews of internet-based psychological treatments for depression such as Internet-based CBT (ICBT) find it an effective alternative to face-to-face psychological treatments with the caveat that guided ICBT is more effective than unguided [47]. Findings also support therapist contact before and/or after ICBT have further efficacious effect of treatment [47]. Concerns toward ICBT include a meta-analysis published in 2013 of effectiveness of computerized CBT on adult depression showing the lack of significant effect of long-term treatment outcomes compared to short-term treatment duration and significantly high participant drop-out [48].

Despite the disadvantages, we believe the above studies give good reason for increased attention to commercial airlines considering work-experience tailored interventions such as ICBT for treating mental health problems, specifically depression, among pilots. Such initiatives could run parallel with leadership endorsement of professional face-to-face contact throughout the guided recovery process. We acknowledge our study does not evaluate how to increase access to treatment and cannot rate or recommend a specific treatment. However, ICBT is one example of a possible intervention found in the literature.

We acknowledge the inability to draw causal inferences due to the study design. However, the numbers raise concern regarding mental health among pilots. Limitations of this study include potential underestimation of frequencies of adverse mental health outcomes due to less participation among participants with more severe depression compared to those with less severe or without depression. This would lead to downward bias of the true estimate of depression prevalence over the survey period. Conversely, upward bias could occur if participants with underlying mental illness are more likely to participate and complete a survey than those without illness due to participant familiarity with the purpose of the study. We believe upward bias is minimized since participants are less likely to know the focus of our study because the survey covers many topics other than depression or suicidal thoughts. In addition, the survey was not described to participants as a mental health study but as a pilot health study.

Furthermore, completers worked as a pilot significantly longer on average than non-completers by over a year and more of them worked in the past 30 days than non-completers. Because of this, completers may exhibit better general health than non-completers and report lower frequency of depressive symptoms. We could not assess this due to non-responses.

Another source of underestimation is the length of the online survey. After implementation, we received feedback regarding the survey being too lengthy. Thus, if survey completers are different in characteristics from non-completers and if this difference influences depression scores, we posit the length of the survey may discourage more depressed participants from completing the survey. This also would result in downward bias.

This study did not conduct clinical interviews of survey respondents to confirm diagnosis of depression, nor did it have access to medical records. We felt the strength of participant anonymity out-weighed the ability to gather this information, and the medical literature provides evidence for good sensitivity and specificity of the PHQ-9 diagnosis compared with diagnosis from structured interviews [26, 28, 29].

Another limitation of this study is reduced generalizability to the general population of airline pilots. This is due to non-random sampling, incomplete participation, and the inability to determine an exact reference population due to anonymous participation. That said, aviation health researchers have utilized anonymous surveying before and published results while acknowledging these same limitations [18]. Furthermore, the only way to achieve responses from airline pilots was to make the survey completely anonymous. Nevertheless, the key findings remain surprising–hundreds of pilots currently flying are managing depression, and even suicidal thoughts, without the possibility of treatment due to the fear of negative career impacts.

## Conclusion

This study fills an important gap of knowledge by providing a current glimpse of mental health among commercial airline pilots, which to date had not been available. Our study found 233 (12.6%) of the 1848 airline pilots responding to the PHQ-9 met criteria for likely depression. Of the 1430 pilots who reported working as an airline pilot in the last seven days at time of survey, 193 (13.5%) met these criteria. Seventy-five participants (4.1%) reported having thoughts of better being off dead or self-harm within the past two weeks. We found a significant trend in proportions of depression at higher levels of use of sleep-aid medication (trend test z = 6.74, *p* < 0.001) and among those experiencing sexual harassment (z = 3.18, *p* = 0.001) or verbal harassment (z = 6.13, *p* < 0.001). Although the results have limited generalizability, there are a significant number of active pilots suffering from depressive symptoms. Future studies will evaluate additional predictors such as sleep and circadian rhythm disturbances.

Poor mental health is an enormous burden to public health worldwide. The tragedy of Germanwings flight 4U 9525 should motivate further research into assessing the issue of pilot mental health. Although current policies aim to improve mental health screening, evaluation, and record keeping, airlines and aviation organizations should increase support for preventative treatment.

## Additional file

Additional file 1: Airline Pilot Mental Health Screening. (DOCX 24 kb)

## Abbreviations

AMEs: Aeromedical examiners
CBT: Cognitive behavioral treatment
CDC-NCHS: U.S. Centers for Disease Control – National Center for Health Statistics
d.f.: Degrees of freedom
DSM-IV: Diagnostic and Statistical Manual of Mental Disorders, Edition 4
FAA: U.S. Department of Transportation, Federal Aviation Administration
ICBT: Internet-based CBT
IP: Internet protocol
IQR: Interquartile range
IRB: Institutional Review Board
MDD: Major depressive disorder
NHANES: U.S. National Health and Nutrition Examination Survey
PHQ-9: Patient Health Questionnaire 9
U.S.: United States
YLDs: Years of life lived with a disability
