# Correction to: Airplane pilot mental health and suicidal thoughts: a cross-sectional descriptive study via anonymous web-based survey

**DOI:** 10.1186/s12940-017-0341-2

**Published:** 2017-11-27

**Authors:** Alexander C. Wu, Deborah Donnelly-McLay, Marc G. Weisskopf, Eileen McNeely, Theresa S. Betancourt, Joseph G. Allen

**Affiliations:** 1 Department of Environmental Health, Harvard T.H. Chan School of Public Health, 665 Huntington Avenue, Building 1, Room 1301, Boston, MA 02115 USA; 2 Department of Global Health and Population, Harvard T.H. Chan School of Public Health, 665 Huntington Avenue, Building 1, Room 1104, Boston, MA 02115 USA; 3 Harvard T.H. Chan School of Public Health, 401 Park Drive, Landmark Center, 404-L, Boston, MA 02215 USA

## Correction

After publication of the article [1], it has been brought to our attention that the specificity of the tool was reported as 88%. This specificity is for Major Depressive Disorder. The specificity of the tool for all depressive disorders, the outcome of interest in the study, is 93%.
