# Supplementary material for: Airplane pilot mental health and suicidal thoughts: a cross-sectional descriptive study via anonymous web-based survey
Source: Environ Health. 2016 Dec 15;15:121. doi: 10.1186/s12940-016-0200-6 (PMC5157081; doi:10.1186/s12940-016-0200-6)
Supplement: Additional file 1: — Airline Pilot Mental Health Screening. (DOCX 24 kb) [file 12940_2016_200_MOESM1_ESM.docx]

**SUPPLEMENTAL: AIRLINE PILOT MENTAL HEALTH SCREENING**

Psychological diagnoses, including depression, and prescription of psychiatric medication can precipitate a pilot losing the FAA Medical Certificate and subsequent loss of license to operate an airplane, which is permanent or temporary depending on diagnosis. Fear of this scenario is a likely reason pilots underreport mental health problems and underutilize professional medical help.[1-4] Airlines perform initial standard screening during the interview process, including self-reporting questions mentioned earlier. Standardized written tests constitute the evaluation. No follow-up screening or testing occurs after employment; only the required FAA First or Second Class medical examinations, which do not screen for mental health (only self-disclosure by pilots). The hiring process at most airlines has the applicant submit an employment application, complete one or several interviews, a written evaluation, complete a simulator proficiency flight, and pass a general physical examination (D. Donnelly-McLay, personal communication, Dec 3, 2015). Once the applicant has accepted employment at an airline, there are no further reviews or screenings by the airline. The only requirement is for the pilots to pass their annual or bi-annual FAA Medical Examination and complete and pass the airline’s annual or semi-annual simulator training.

In the United States, all airline pilots are required to undergo an annual or bi-annual FAA Medical Examination. There is no mental health screening requirement or psychological testing during these exams. Pilots complete the FAA Form 8500-8 Medical Certificate, a self-report. Mandatory response questions include:

*“Have you ever in your life been diagnosed with, had, or presently have any of the following: Mental disorders of any sort, depression, anxiety, etc.; Suicide attempt; Alcohol dependency or abuse; Substance dependence or a failed drug test ever; or substance abuse or use of illegal substance in the last two years?”*

Current requirements for these exams performed by an Aeromedical Examiner (AME) prohibit, “diagnosis of psychosis, bipolar disorder, or severe personality disorder.”[5] Any mental health issue is only identified by self-report. In the case of a pilot who self-reports having depression, the AME would report the pilot as unfit to fly unless the AME, “…*considers the applicant’s condition as unlikely to interfere with the safe exercise of the applicant’s licence and rating privileges*” (*9.1.3 Annex 1, 6.3.2.2.1).*[6]

Europe and other worldwide regions follow International Civil Aviation Organization (ICAO) guidelines from the *ICAO Manual of Civil Aviation Medicine*, which identify mental health concerns but leaves out specifics of verifying diagnoses.[6] A medical examiner cannot issue a passing medical exam if the pilot admits to having any type of diagnosed mental disorder. The manual recommends a pilot is unfit to fly if diagnosed with depression and is currently using anti-depressant medication. Since 2012, the ICAO notes mental health issues merit increased attention. ICAO proposes the requirement to implement a health education system for holders of pilot licenses. If approved by the ICAO Air Navigation Commission, this is one step forward in addressing pilot mental health.[7]

Self-reported medical conditions that chronically interfere with sleep such as insomnia, sleep apnea, or depression/anxiety disqualify a pilot for aviation duties.[5] The FAA permits “occasional or limited use” of sleep aids and prohibits “daily/nightly use.”[5] A list of acceptable sleep aids and minimum required wait time prior to flying indicate at least 24 hours to 72 hours.[8]

**Aviation Medical Examiner**

The role of the Aviation Medical Examiner is to “examine applicants and determine whether or not they meet the medical standards for the issuance of the airman medical certificate…The medical standards are found in Title 14 of the Code of Federal Regulations, Part 67.”[5] The regulations pertaining to the medical standards of mental health are:

I. Code of Federal Regulations All Classes: 14 CFR 67.107(a)(b)(c), 67.207(a)(b)(c), and 67.307(a)(b)(c) (a) No established medical history or clinical diagnosis of any of the following: (1) A personality disorder that is severe enough to have repeatedly manifested itself by overt acts. (2) A psychosis. As used in this section, "psychosis" refers to a mental disorder in which: (i) The individual has manifested delusions, hallucinations, grossly bizarre or disorganized behavior, or other commonly accepted symptoms of this condition; or (ii) The individual may reasonably be expected to manifest delusions, hallucinations, grossly bizarre or disorganized behavior, or other commonly accepted symptoms of this condition. (3) A bipolar disorder.[9]

**Additional Pilot Testing**

Airline pilots are also subject to random drug and alcohol testing throughout their careers, although this testing is not part of the annual FAA Medical examinations. This Testing is required by the Omnibus Transportation Employees Testing Act of 1991 and by DOT and FAA regulations (49 CFR part 40 and 14 CFR part 120).[10]

**REFERENCES**

1 Parker PE, Stepp RJ, Snyder QC. Morbidity among airline pilots: the AMAS experience. Aviation, Space, and Environmental Medicine. 2001;72:816-20.

2 Lollis BD, Marsh RW, Sowin TW, Thompson WT. Major depressive disorder in military aviators: a retrospective study of prevalence. Aviation, Space, and Environmental Medicine. 2009;80:734-37.

3 Aviation Today’s Daily Brief. Pilots on prozac. Aviation Today's Daily Brief. 2010;2.

4 Aviation Medicine Advisory Service. Counseling, depression and psychological support. 2016. https://www.aviationmedicine.com/article/counseling-depression-and-psychological-support/. Accessed 22 Feb 2016.

5 Federal Aviation Administration. Guide for aviation medical examiners. 2015. https://www.faa.gov/about/office_org/headquarters_offices/avs/offices/aam/ame/guide/. Accessed 9 Dec 2015.

6 International Civil Aviation Organization. ICAO manual of civil aviation medicine, 3rd Ed., 2012. http://www.icao.int/publications/Documents/8984_cons_en.pdf. Accessed 9 Dec 2015.

7 International Civil Aviation Organization. Working Paper AN-WP/8927: Preliminary review of proposed amendments to annex 1 arising from MPSG relating to health education and the medical assessment process, and safety management principles as applied to the medical assessment process. 2015.

8 Federal Aviation Administration. Guide for aviation medical examiners: Pharmaceuticals (therapeutic medications) sleep aids. 2015. http://www.faa.gov/about/office_org/headquarters_offices/avs/offices/aam/ame/guide/pharm/sleepaids/. Accessed 11 Mar 2016.

9 U.S. Government Publishing Office. 14 CFR 67 – Medical standards and certification. 2012. http://www.gpo.gov/fdsys/pkg/CFR-2012-title14-vol2/pdf/CFR-2012-title14-vol2-part67.pdf. Accessed 11 Mar 2016.

10 Federal Aviation Administration. Industry drug and alcohol testing program. 2015. https://www.faa.gov/about/office_org/headquarters_offices/avs/offices/aam/drug_alcohol/. Accessed 11 Mar 2016.
